# 22732 Impact of Type-I Interferon Manipulation During Embryo Implantation and Placentation

**DOI:** 10.1017/cts.2021.644

**Published:** 2021-03-31

**Authors:** Michael K. Simoni, Sydnie Swanson, Monica Mainigi, Kellie Jurado

**Affiliations:** University of Pennsylvania

## Abstract

ABSTRACT IMPACT: This research will promote understanding the role of the Type-I Interferon signaling pathway during embryo implantation, potentially leading to a new diagnostic or treatment target in early pregnancy failure. OBJECTIVES/GOALS: Studies suggest interferon signaling regulation is tightly balanced between physiologic and pathophysiologic growth in early pregnancy. We propose to determine the impact of interferon-mediated inflammation on embryo implantation and early pregnancy failure in normal conditions and chronic inflammatory diseases in a novel mixed-mouse model. METHODS/STUDY POPULATION: To probe the role of type-I interferons (IFNs) in implantation, we will utilize a mouse model and non-surgically transfer both Ifnar1-/- and Ifnar1-/+ embryos into an immune-competent pseudopregnant wild-type female recipient. This will allow analysis of a litter with distinct genotypes within the same, immune-competent, uterine environment. Type-I IFN stimulation will be systemically induced with Poly-(I:C) at various time points around implantation. A similar approach will be used in mouse models of chronic inflammatory disease states associated with early pregnancy loss (e.g. systemic lupous erythematous). With this model, we will be able to control for deficiencies in maternal immune response to specifically determine the embryonic response to inflammation during implantation and development. RESULTS/ANTICIPATED RESULTS: We anticipate the Ifnar1-/+ embryos - those able to respond to Type-I IFN - and their surrounding implantation sites will exhibit more maternal-fetal barrier dysfunction in the form of impaired trophoblast fusion, improper formation of the microvascular architecture, and increased permeability of the maternal-fetal barrier, compared to embryos unable to respond to IFN. We will also conduct similar analyses in mouse models of chronic inflammatory diseases. We hypothesize these mice to have baseline endometrial inflammation that stimulated the IFN-pathway in IFN-capable embryos, producing breakdown of the maternal-fetal barrier. In these mice, we predict Ifnar1-/- embryos will show improved molecular outcomes when compared to Ifnar1-/+ embryos, and thus improved associated pregnancy outcomes. DISCUSSION/SIGNIFICANCE OF FINDINGS: This work can insight into the immunological mechanisms that govern embryo implantation and early placentation. This could provide more pointed means for management and intervention of early pregnancy failure and/or disease states that are commonly associated with poor reproductive outcomes.

